# The gamma rhythm as a guardian of brain health

**DOI:** 10.7554/eLife.100238

**Published:** 2024-11-20

**Authors:** Ana Maria Ichim, Harald Barzan, Vasile Vlad Moca, Adriana Nagy-Dabacan, Andrei Ciuparu, Adela Hapca, Koen Vervaeke, Raul Cristian Muresan

**Affiliations:** 1 Transylvanian Institute of Neuroscience, Department of Experimental and Theoretical Neuroscience Cluj-Napoca Romania; 2 Preclinical MRI Center, Interdisciplinary Research Institute on Bio-Nano-Sciences, Babeș-Bolyai University Cluj-Napoca Romania; 3 Faculty of Biology and Geology, Babeș-Bolyai University Cluj-Napoca Romania; 4 Department of Molecular Medicine, Institute of Basic Medical Sciences, University of Oslo Oslo Norway; 5 STAR-UBB Institute, Babeș-Bolyai University Cluj-Napoca Romania; Indian Institute of Science Bangalore India; University of Texas at Austin United States

**Keywords:** gamma oscillations, GENUS, homeostasis, cortex, interneurons

## Abstract

Gamma oscillations in brain activity (30–150 Hz) have been studied for over 80 years. Although in the past three decades significant progress has been made to try to understand their functional role, a definitive answer regarding their causal implication in perception, cognition, and behavior still lies ahead of us. Here, we first review the basic neural mechanisms that give rise to gamma oscillations and then focus on two main pillars of exploration. The first pillar examines the major theories regarding their functional role in information processing in the brain, also highlighting critical viewpoints. The second pillar reviews a novel research direction that proposes a therapeutic role for gamma oscillations, namely the gamma entrainment using sensory stimulation (GENUS). We extensively discuss both the positive findings and the issues regarding reproducibility of GENUS. Going beyond the functional and therapeutic role of gamma, we propose a third pillar of exploration, where gamma, generated endogenously by cortical circuits, is essential for maintenance of healthy circuit function. We propose that four classes of interneurons, namely those expressing parvalbumin (PV), vasointestinal peptide (VIP), somatostatin (SST), and nitric oxide synthase (NOS) take advantage of endogenous gamma to perform active vasomotor control that maintains homeostasis in the neuronal tissue. According to this hypothesis, which we call GAMER (GAmma MEdiated ciRcuit maintenance), gamma oscillations act as a ‘servicing’ rhythm that enables efficient translation of neural activity into vascular responses that are essential for optimal neurometabolic processes. GAMER is an extension of GENUS, where endogenous rather than entrained gamma plays a fundamental role. Finally, we propose several critical experiments to test the GAMER hypothesis.

## Introduction

Gamma oscillations are rhythmic modulations of brain activity, with typical frequencies in the range of 30 to 120–150 Hz. The exact definition of the frequency band varies across studies, with some restricting it to 30–90 Hz (Buzsáki and Wang, 2012), while others extending it to 30–120 Hz (Tallon-Baudry, 2009), or even 30–150 Hz (Fernandez-Ruiz et al., 2023). Despite being studied for over eight decades now (Jasper and Andrews, 1938), gamma oscillations remain a mysterious brain rhythm whose functional role is yet to be fully understood (Sedley and Cunningham, 2013). Compared to the other brain rhythms, gamma is relatively fast, the gamma cycle’s timescale (7–33ms) matching relevant time constants of neuronal membranes (Fries et al., 2007) and the time windows that are important for synaptic plasticity (Bi and Poo, 1998). Therefore, it has been suggested numerous times that gamma oscillations are important for perceptual (Tiesinga and Sejnowski, 2009; Yang et al., 2022), cognitive (Başar, 2013), and behavioral processes (Anand et al., 2023; Gupta and Chen, 2016; Schoffelen et al., 2011).

Studying gamma oscillations in brain signals is a difficult endeavor, for several reasons. First, there is confusing terminology in the field, where ‘gamma’ is used to term different phenomena. Gamma band activity should not be confused with gamma oscillations. While the former refers to any signal whose spectral signature falls within the gamma frequency range, this does not necessarily identify an oscillation (Buzsáki and Wang, 2012). For example, a ‘saccadic spike potential’ in the electroencephalogram (EEG), associated with visual miniature microsaccades, has a typical broadband signature that falls within the gamma range (Yuval-Greenberg et al., 2008), but this is not an oscillation, the signal lacking periodicity. By contrast, we (Mureşan et al., 2008) and others (Buzsáki and Wang, 2012), define gamma oscillations as a periodic signal modulation, usually confined to a narrow region of the gamma band (Ardelean et al., 2023).

**Figure 1.**
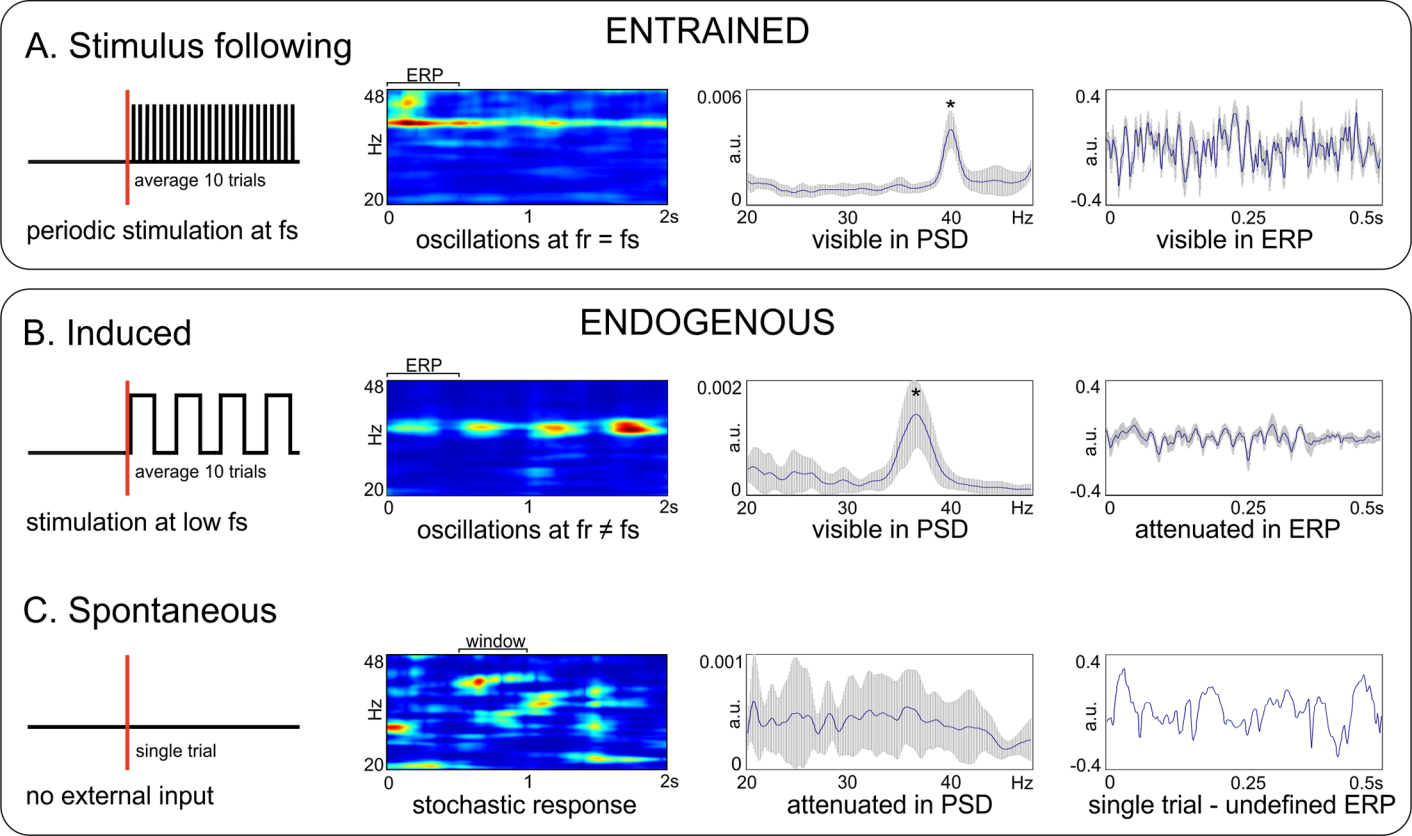
Terminology relating gamma oscillations to stimulation. Top box: Entrained oscillations do not require an internal generation mechanism, but simply activate in response to a periodic stimulation. (**A**) Entrained oscillations follow closely the stimulation frequency. Bottom box: Endogenous oscillations rely on the existence of an internal oscillation-generating mechanism. (**B**) Induced oscillations. (**C**) Spontaneous oscillations. From left to right: diagram of experimental design and stimulus delivery pattern (sketch); time-frequency representation (TFR); power spectral density (PSD; * - marks a visible peak); event-related potentials (ERP) for the trial segment marked on the spectrogram. Data was acquired from V1 of awake C57BL/6 mice during light flicker stimulation at 40 Hz (in panel A), and anesthetized C57BL/6 mice during presentation of oriented drifting gratings (panel B) and during absence of stimulation (panel C). Measures of amplitude and power are computed on z-scored normalized data. *f_s_* – stimulation frequency; *f_r_* – frequency of the response. Error bands represent s.d.

Another terminological issue pertains to the way gamma oscillations relate to the external stimulation (or the lack of it). This is essential when multiple trials are recorded and frequency- as well as time-domain analyses are performed. In general, evoked oscillations occur always at a precise phase relative to the stimulus timing, in each recorded trial. When the stimulus is also periodic, this is called entrainment (Figure 1A), that is the oscillation follows closely the rhythmic input. The oscillation is clearly visible in the time-frequency representation (TFR) as a region of elevated power, and in the power-spectral density (PSD; also called spectrum) as a distinct peak at the stimulation frequency. Importantly, generalized, m-to-n locking is also possible between the internal dynamics and stimulus, such that the ratio of the two frequencies can be a rational number (m/n; Rosenblum et al., 2001; Tass et al., 1998). For example, oscillations could be entrained at 20 Hz with 40 Hz stimulation. Moreover, when signals are averaged over trials, aligned to the onset of stimulation, one obtains an event-related potential (ERP) with large amplitude and clear sign of periodic modulation, aligned to the entraining stimulus (or to multiples of its period for m-to-n locking). By contrast, induced gamma oscillations (Figure 1B) are generated by internal circuit mechanisms when the stimulus is either aperiodic or has a significantly lower frequency. For example, the periodic passage of white bars of a drifting grating through the receptive fields of neurons from primary visual cortex generates bursts of gamma oscillations at much higher frequency than the temporal frequency of the grating (Figure 1B). In this case, the oscillation is still visible in the frequency domain (TFR and PSD) but its frequency does not match the slow stimulation frequency of the grating. In addition, in each trial the phase of the gamma oscillation is unrelated to the timing of the stimulus, such that the ERP amplitude is much diminished. For a large number of trials, the ERP will typically not display any modulation at the gamma frequency. Finally, we define spontaneous gamma (Figure 1C), as produced in the absence of any external stimulus during ongoing, ‘spontaneous’ brain activity. In such cases, ‘single trial’ analyses (analyzing a single time trace) can reveal robust gamma bursting, at different frequencies and timepoints (Figure 1C), but this may not be readily visible in the PSD. Therefore, the lack of a clear bump in the spectrum (PSD) does not indicate the absence of robust gamma bursting in the data. In addition, if multiple spontaneous time-traces were averaged, the average (similar to an ERP) would also not show any clear component and would exhibit low amplitude.

In the context of this review, we will call every type of gamma oscillation that is not entrained as ‘endogenous’. Such oscillations necessarily need an internal, endogenous circuit mechanism that generates them. By contrast, entrained gamma does not require the existence of internal generating mechanisms—it is sufficient for the system to follow the external rhythm provided as input.

**Figure 2.**
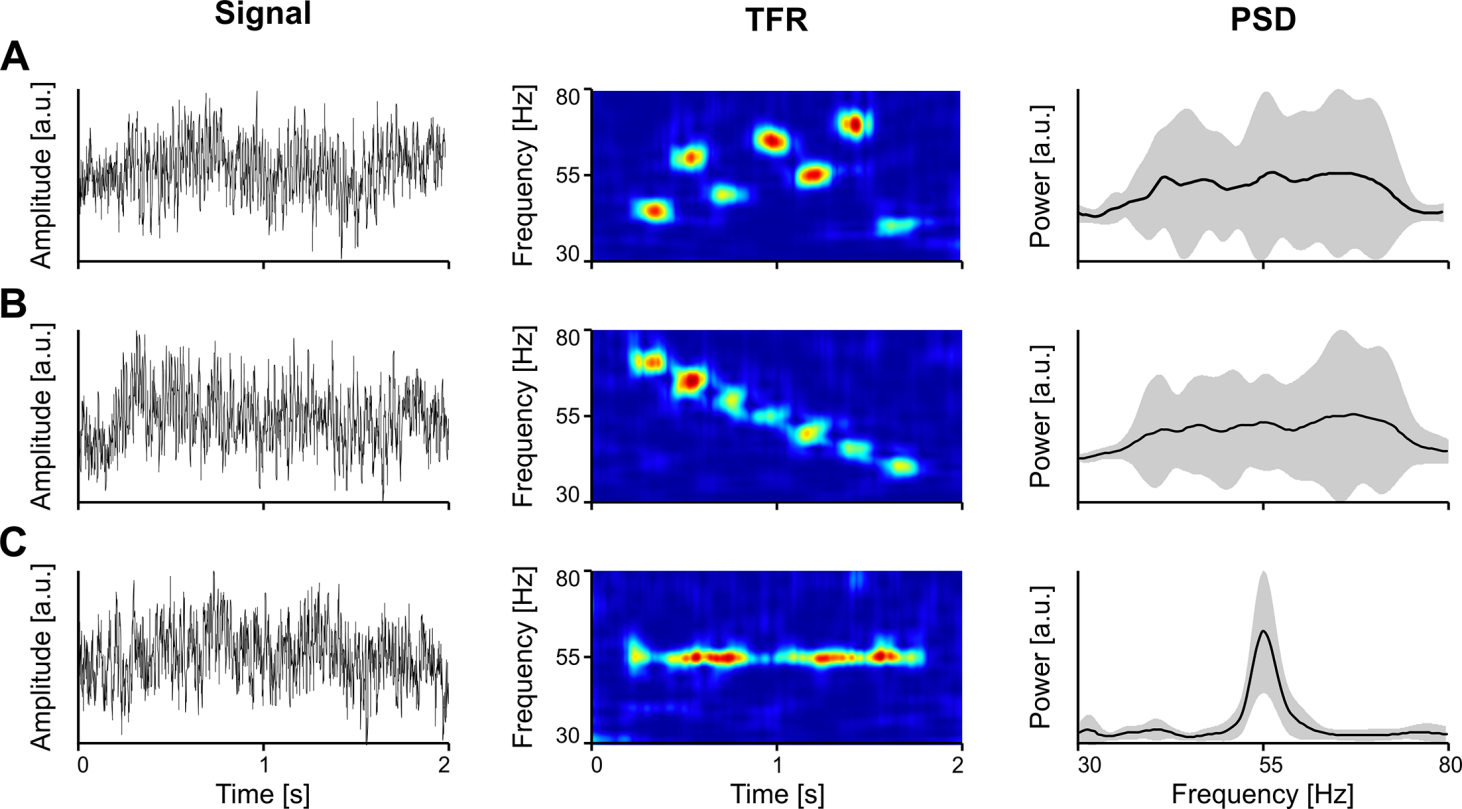
The spectrum (PSD) does not always reveal the presence of gamma oscillations in the signal. (**A**) Vigorous gamma bursting across the time-frequency landscape. (**B**) Succession of bursts with linearly decreasing frequency. (**C**) Induced oscillation around 55 Hz. Toy data generated by inserting oscillatiory packets (Morlet atoms) into a pink noise signal. Error bands represent s.d. TFR, time-frequency representation; PSD, power spectral density.

Importantly, while narrowband gamma oscillations, with a clear bump in the power spectrum may be contingent upon specific conditions, such as stimulus intensity, stimulus properties (e.g. visual contrast), or behavioral state of the animal (Saleem et al., 2017), the lack of a bump in the spectrum does not indicate the absence of gamma. Only TFRs, such as the spectrogram or scalogram, can reveal the true expression of gamma oscillations, exposing the underlying bursting process. In Figure 2, we show examples where the spectrum (PSD) does not clearly reflect the underlying bursting gamma process, when bursts are scattered in time and frequency (Figure 2A) or when systematic bursts at different frequencies overlap along the frequency domain, abolishing a clear peak (Figure 2B). In order for the PSD to show a robust peak, the oscillation needs to be narrow-band, with a significant power increase relative to the background, and sustained at a stable frequency for long enough in the analysis window (Figure 2C).

To correctly assess the expression of gamma in a certain analysis window, it is critical to avoid relying on the PSD and to use TFRs. However, the latter are also posing significant challenges. There has been increasing awareness in the past decade that, unlike other rhythms, gamma does not arise as a continuous, sustained process, over long periods of time. Instead, gamma is typically produced by circuit generators (Fernandez-Ruiz et al., 2023), occurring as brief oscillation bursts (Burns et al., 2011; Burns et al., 2010; Tal et al., 2020), also called ‘packets’ (Moca et al., 2021). While in the averaged (across trials) TFR gamma could mimic a continuous band of increased power, over hundreds of milliseconds, single trial analysis frequently reveals that it emerges from sparse, disparate bursting, with each burst narrowly confined in time and frequency (Tal et al., 2020). This raises a methodological issue: Due to their finite nature, gamma bursts are difficult to localize simultaneously in time and frequency. Traditional analysis techniques relying on the Fourier transform or single wavelets (Le Van Quyen and Bragin, 2007) are suboptimal, masking the expression of gamma bursts in complex neural signals.

The above-mentioned difficulties in detecting and correctly characterizing gamma bursts as events produced by gamma generators Fernandez-Ruiz et al., 2023 have at least slowed down, if not hindered progress in elucidating gamma’s functional role. Fortunately, other spectral estimation techniques exist. For example, matching pursuit (MP; Durka and Blinowska, 1995) is an innovative approach for time-frequency representation, in which the signal is decomposed in atoms (localized oscillations) chosen from a very large dictionary, which can be tailored to shapes expected to be found in the signal. The method was used successfully to reveal longer oscillatory bursts than with Fourier or wavelets (Chandran Ks et al., 2018). Unfortunately MP does not cope well with abrupt changes in the oscillation power (Chandran Ks et al., 2018), such as those observed for gamma (Burns et al., 2011; Burns et al., 2010; Tal et al., 2020). More recent time-frequency super-resolution techniques, such as the superlets (Moca et al., 2021), do not suffer from this issue, although they may have other shortcomings compared to MP. Superlets are able to precisely detect and localize gamma bursts (Ardelean et al., 2023). Novel, robust time-frequency estimation techniques are expected to be a game-changer, opening a new window on a yet insufficiently explored world.

Recently, a procedure that is being actively researched and developed as a non-invasive therapy for Alzheimer’s disease, may suggest a surprising and overlooked functional role for gamma oscillations. The procedure, called Gamma ENtrainment Using Sensory stimulation (GENUS) (Iaccarino et al., 2016), attempts to entrain brain oscillations at 40 Hz by delivering periodic stimuli (flicker) to different sensory modalities: visual, auditory, or tactile. This has been further expanded to direct brain stimulation using transcranial alternating current stimulation—tACS (Dhaynaut et al., 2022; Kim et al., 2021), transcranial magnetic stimulation—TMS (Liu et al., 2022), and near infrared light—NIR (Zomorrodi et al., 2019).

While research is still ongoing on the effectiveness of GENUS for treating Alzheimer’s, we would like to point out that there are other, equally important discoveries, which spin out from this research and whose importance is apparently not yet fully recognized. Causal entrainment or induction of gamma oscillations in brain circuits has been recently shown to correlate with increased blood flow, activation of the glymphatic system, and efficient functional coupling with microglia (Murdock et al., 2024). Moreover, hemodynamic signals in the brain are tightly correlated with gamma oscillations in cortex, and this has been known already since 2005 (Niessing et al., 2005).

Inspired by GENUS, a tantalizing hypothesis emerges that gamma oscillations in brain circuits may have a causal role in the maintenance of healthy brain function by promoting neuroglial coupling and leveraging vascular mechanisms. Here, we propose that endogenous gamma, generated by brain circuits, acts also as a ‘service rhythm’ that regulates healthy blood and glymphatic flow and maintains a ‘hot’ interface with microglia. We challenge the field to causally study if the breakdown of gamma in certain brain disorders is not only the result, but also part of the cause that leads to neural degeneration and circuit dysfunction.

This review offers a structured exploration of gamma oscillations, which we consider not an epiphenomenon, but potentially fundamental to healthy brain function. The first section focuses on the underlying mechanisms responsible for generating and modulating gamma oscillations within neural circuits. Subsequently, it briefly examines the involvement of gamma oscillations in various processes, including cognition, perception, and behavior. Within this section, we discuss the most prominent theories supporting gamma’s functional role, alongside with experimental evidence that both supports and challenges these hypotheses. Following this, we explore the potential of gamma oscillations as a therapeutic tool in treating neurological disorders, such as Alzheimer’s disease (AD). In particular, we present the latest promising non-invasive treatment, GENUS, in both animal models and humans. Finally, we propose a novel hypothesis, that could expand the current understanding on the function of gamma oscillations.

## Mechanisms of endogenous gamma oscillations

We will first review the known mechanisms of emergence of gamma oscillations to further guide mechanistic inferences about their role in brain function. Since their discovery (Berger, 1929), oscillations have been studied and classified primarily based on their central frequencies and gamma is no exception (Jasper and Andrews, 1938). Recent reviews have pointed out that frequency alone, without considering the underlying generators, offers limited opportunities to study gamma for two main reasons (Buzsáki and Wang, 2012; Fernandez-Ruiz et al., 2023). First, bursts of gamma oscillations (Moca et al., 2021) are generated in relatively small local circuits (Buzsaki, 2006; Gray and Singer, 1989; Sirota et al., 2008), usually in the superficial layers (Mendoza-Halliday et al., 2024; Smith et al., 2013; Xing et al., 2012b). Therefore, because gamma is much more localized in time and space and because multiple generators coexist, a single readout electrode can easily pick up gamma from multiple sources that cannot always be distinguished solely by the temporal pattern or frequency. Second, gamma oscillations injected in the LFP by synapses from distant neurons blur even more the contribution of local circuits to observed gamma (Fernandez-Ruiz et al., 2023). Overall, there is compelling evidence that local generators and distant transmission of gamma oscillations should be taken into account whenever possible in order to dissect and study gamma (Buzsáki and Wang, 2012; Fernandez-Ruiz et al., 2023). For instance in mice, during learning, the dentate gyrus exchanges spatial- and object-related information with the medial and lateral entorhinal cortex using high- (100–150 Hz) and low-frequency gamma (30–50 Hz), respectively (Fernández-Ruiz et al., 2021).

Currently, it is well accepted that fast-spiking, parvalbumin (PV) positive interneurons are instrumental to the generation of the gamma rhythm (Buzsáki and Wang, 2012; Fernandez-Ruiz et al., 2023; Kriener et al., 2022; Tiesinga and Sejnowski, 2009). Historically, proof about the involvement of interneurons in gamma generation first came from pharmacological interventions (Whittington et al., 1995), which was later confirmed by optogenetic studies. The latter studies, by Cardin and colleagues, showed that rhythmic stimulation of targeted fast-spiking interneurons in the barrel cortex selectively enhances gamma at the stimulation frequency (Cardin et al., 2009). At the same time, it was also shown that diffuse activation and inactivation of PV^+^ interneurons enhances and suppresses gamma, respectively (Sohal et al., 2009).

In the early 2000s, theoretical studies on the origins of gamma have put forth two mechanisms by which gamma oscillations can arise in local circuits (Börgers and Kopell, 2003; Whittington et al., 2000) and, in both, inhibitory neurons play a crucial role. First, in a network of purely inhibitory neurons, it has been shown that recurrent connections are enough to synchronize the network and produce oscillations. There is, of course, a prerequisite of enough excitatory drive to produce neuronal spiking, but other than that, oscillations occur naturally. This mechanism is known as Interneuron Network Gamma (ING; Whittington et al., 2000). The second theoretical mechanism is the Pyramidal-Interneuron Network Gamma (PING; Börgers and Kopell, 2003; Whittington et al., 2000). As opposed to ING, in PING both excitatory and inhibitory neurons are required. Here, oscillations are a consequence of the push-pull between excitation and inhibition. The buildup of drive within the excitatory population results in an excitatory volley that activates the inhibitory population, which in turn quenches the circuit with an inhibitory volley. In a network, this repeated push-pull interaction gives rise to oscillations (Hansel and Mato, 2003). It is not yet clear whether ING and PING coexist or compete in vivo (Tiesinga and Sejnowski, 2009). However, many studies have observed experimentally the succession of excitation-inhibition volleys characteristic of PING (Bragin et al., 1995; Hansel and Mato, 2003; Le Van Quyen and Bragin, 2007; Tiesinga and Sejnowski, 2009).

In both ING and PING, the frequency of gamma oscillations depends on the strength of the input and on synaptic delays. Scaling the drive up and down modulates the frequency within the circuit. Interestingly, it has been shown that membrane resonance, especially when expressed in interneurons, promotes stable oscillation frequency (Moca et al., 2014), decoupled from input strength fluctuations, as reported experimentally (Gray and Singer, 1989; Moca et al., 2014). Therefore, a third mechanism contributing to gamma has been defined, called Resonance INduced Gamma (RING). Stabilization of frequency by resonance could be a dynamic phenomenon because resonance can be regulated by membrane voltage and neuromodulators (Hutcheon et al., 1996; Steriade et al., 1991), and thus it could be subject to cortical-state changes and be controlled by top-down feedback loops (Moca et al., 2014).

Gamma oscillations are not restricted to isolated local circuits (Buzsáki and Wang, 2012). Larger networks that exchange and mix gamma oscillations have been found in hippocampus and cortex. For example, a well-known circuit in hippocampus formed by CA3, the entorhinal cortex, and CA1 is summarized in Fernandez-Ruiz et al., 2023. In short, CA1 receives low-frequency gamma oscillations (30–50 Hz) produced by CA3 and higher frequency (60–100 Hz) oscillations from the entorhinal cortex. At the same time, CA1 produces its own gamma at even higher frequency (100–150 Hz). In this particular case, the three gamma rhythms in CA1 of different origins are segregated in frequency and in time, as their peak power aligns to different phases of the theta oscillation. In cortex, multiple gamma generators have been described, each with their own particularities (Fernandez-Ruiz et al., 2023) and with laminar specificity (Senzai et al., 2019). In mouse visual cortex under anesthesia, while stimulus induced gamma is visible in layers 2–4, spontaneous gamma is present across all layers (Welle and Contreras, 2016).

Primary visual cortices across different species differ in their response properties and mechanisms underlying gamma (Han et al., 2022). For instance, in macaques dark stimuli induce an increase in gamma power (Xing et al., 2014), whereas in mouse visual cortex gamma activity is predominantly triggered by bright stimuli. In addition, the origin of gamma activity may also differ across species. While in macaques gamma can be generated cortically, in the absence of LGN gamma (Bastos et al., 2014), in mice gamma involves pre-cortical structures, including LGN and retina (Saleem et al., 2017; Storchi et al., 2017). Additionally, a study by Castelo-Branco et al., 1998 found two distinct narrow gamma peaks in cat visual cortex having cortical and subcortical sources. Overall, the literature shows that distinct mechanisms and sources may mix and match to generate the electrophysiological observables at the electrode site and that care must be taken when translating across species.

## Gamma oscillations in perception, cognition, and behavior

We will next briefly discuss some of the proposed roles of gamma in brain function and the associated controversies. For more details, please also see Han et al., 2022.

### Functional role of gamma?

Gamma oscillations have been extensively documented in a wide range of cortical and subcortical areas in mammals, including visual (Hermes et al., 2015a), somatosensory (Gross et al., 2007), auditory (Steinschneider et al., 2008), motor (Muthukumaraswamy, 2010), and frontoparietal brain regions (Castellano et al., 2014). They have been also observed in several subcortical areas such as amygdala (Popescu et al., 2009), hippocampus (Colgin and Moser, 2010), thalamus (Minlebaev et al., 2011), superior colliculus (Le et al., 2019), cerebellum (Middleton et al., 2008), and others.

While the mechanisms of gamma and its role across species are still not fully elucidated (Han et al., 2022), it is however important to note that, in addition to well-known model species, such as monkeys and rodents, gamma has also been observed in the olfactory bulb and pyriform cortex of hedgehogs (Adrian, 1942), in the optic lobes of flies (Grabowska et al., 2020), in the olfactory lobe of locusts (Laurent and Davidowitz, 1994), rabbits (Rojas-Líbano and Kay, 2008), and in the optic tectum of birds (Lewandowski and Schmidt, 2011).

There is mounting evidence that gamma is not simply a byproduct of neural activity (i.e. just a natural phenomenon arising from the balance of excitation and inhibition) but that its expression may facilitate useful computations. It has been suggested that gamma increases signal discrimination (Masuda and Doiron, 2007), enhances the efficacy and efficiency of signal transmission (Knoblich et al., 2010), contributes causally to attention (Kim et al., 2016), mediates interactions between hippocampus and cortex (Colgin et al., 2009; Pedrosa et al., 2022) facilitating selection of experience for memory (Yang et al., 2024) via hippocampal sharp wave ripples (Pochinok et al., 2024), and so on. In addition, gamma is known to be disrupted in brain disorders (Mably and Colgin, 2018; Paterno et al., 2021; Uhlhaas and Singer, 2006).

An EEG study by Murty et al., 2020 on healthy elderly subjects (50–88 years) has shown that there is an inverse correlation between the power of gamma and aging . This effect was more pronounced for fast gamma. Additionally, in a follow up study, they found that stimulus-induced gamma activity was significantly lower in subjects with mild cognitive impairment (MCI) and AD compared to their healthy counterparts (Murty et al., 2021). Similar findings have also been observed in motor areas of autistic spectrum disorder subjects (Gaetz et al., 2020). The number of studies ascribing putative functional roles to gamma oscillations is actually very large, but direct, causal evidence for the essential role of gamma in healthy brain activity is still relatively sparse.

The ubiquity of gamma oscillations across species and cortical areas has prompted the development of at least three important hypotheses. One of the most prominent ones is the Binding by Synchrony hypothesis (Singer, 2007; Singer, 1999), which proposes that disparate features of a sensory representation are ‘bound’ into coherent percepts by subpopulations of neurons that synchronize their spiking activity (Singer, 2007). Gamma oscillations may serve as an active process that synchronizes neurons into specific constellations, aligned to the gamma cycles.

Another hypothesis, known as Communication through Coherence (CTC), underscores the importance of coherence as a mechanism for information transfer across different brain areas. The CTC hypothesis posits that effective cortical communication relies on rhythmic synchronization within and between pre- and postsynaptic neuronal groups (Fries, 2005). When activated, synchronized neurons alternate between high and low excitability phases, that either enhance or reduce the effect of the synaptic input. If spike outputs are not synchronized in a precise temporal window, the synaptic input arrives ‘out of sync’ leading to a less efficient cortical communication. Experimental evidence supporting the CTC hypothesis includes studies on macaques, where virtually induced gamma synchronization between two primary visual areas (V1 and V4) facilitated sensory transmission and reduced reaction times to motor tasks (Roberts et al., 2013).

The original version of CTC hypothesis, however, has faced some vulnerabilities due to its assumptions of zero-phase synchronization and sinusoidal phase-excitability relations during rhythmic synchronization (Fries, 2005). In fact, recent findings revealed that distant neuronal groups can exhibit high gamma band coherence with systematic delays (Bastos et al., 2015; Grothe et al., 2012). A revised formulation of CTC incorporates non-sinusoidal and non-linear excitability modulation, aligning with mathematical models (Börgers and Kopell, 2008), and integrates entrainment with delay as a necessary mechanism that sets up phase relations during CTC in both unidirectional and bidirectional communication (Fries, 2015).

A third theoretical concept for explaining the functional role of gamma is Phase Coding—PC (Fries et al., 2007). This relies on the concepts of windows of opportunity defined by CTC but explores the non-binary nature of spiking probability as a metric for coding stimulus features. Specifically, PC relies on the fact that inhibition is strongest at the peak and decreases towards the trough of the gamma cycle, as measured extracellularly. Stronger excitatory drive can overcome a higher level of inhibition and therefore can trigger action potentials earlier in the gamma cycle compared to weak drive. Therefore, excitatory strength is encoded in the phase relationship between spikes and the gamma cycle. Phase coding has been discussed since the early 1990s (Buzsáki and Chrobak, 1995) and has further gained popularity due to the discovery of theta-phase precession in the hippocampus and entorhinal cortex for place cells (Hafting et al., 2008; O’Keefe and Recce, 1993; Yamaguchi et al., 2007). In the gamma range, phase coding has received a lot of theoretical backing via modeling studies (Lowet et al., 2015), but direct experimental evidence is difficult to obtain due to the difficult task of precisely assessing the phase of such high frequency oscillations.

### Useful feature or side effect?

Skeptics on the functional role of gamma mainly focus on the hypothesis of binding by synchrony (Singer, 1999), CTC (Fries, 2005), or PC (Fries et al., 2007) and anchor their arguments in three major directions (Ray and Maunsell, 2015): low and inconsistent power, dependence on low-level stimulus features, and phase disruption due to conduction delays and broad-band contamination.

#### Low and inconsistent power

The transient nature of gamma oscillations (Ardelean et al., 2023; Burns et al., 2011; Moca et al., 2021; Tal et al., 2020; Xing et al., 2012a), coupled with their relatively low power as compared to other bands (Henrie and Shapley, 2005; Jia et al., 2011), has brought under question their usefulness in information routing and transmission (Ray and Maunsell, 2015), as postulated by CTC (Fries, 2005) and PC (Fries et al., 2007). The low-power criticism rests on two shoulders. First, the authors (Ray and Maunsell, 2015) argue that in many cases gamma oscillations are so weak that a clear power is visible only after baseline correction (Henrie and Shapley, 2005), but not in the raw spectra. As such, neurons would need to filter or use a threshold in order to detect gamma in their internal dynamics or in the external input. However, this need not be the case. Theoretical studies have shown that the network mechanisms behind gamma (Börgers and Kopell, 2003; Whittington et al., 2000) can easily synchronize networks, while membrane resonance of interneurons can adjust the timing of the firing such that neurons are able to consume gamma without filtering or integration (Moca et al., 2014).

The transient burst-like nature of oscillations has been emphasized recently (Ardelean et al., 2023; Moca et al., 2021; Tal et al., 2020). Because of this, it has been suggested that gamma cannot establish a stable clock (Ray and Maunsell, 2015) limiting its usefulness for communication between sites. In addition, if gamma were useful for transmission, then one would expect dominant gamma in input layer L4, while in reality, gamma is in fact stronger in layers L2-3 (Mendoza-Halliday et al., 2024; Smith et al., 2013; Xing et al., 2012b). This critique refers mainly to the narrow-band, stimulus induced gamma. Gamma, however, is produced by multiple sources, some within the local circuit and some propagating from distant sites via synaptic transmission (Fernandez-Ruiz et al., 2023). By analogy to sparse coding, which is considered beneficial for encoding of information (Gupta and Stopfer, 2014; Olshausen and Field, 1996), sparse gamma could also be functionally relevant. We can speculate that gamma need not be active all the time but bursts of gamma can reflect local computations, synaptic input, or could be used for transient coupling, while the selective absence of gamma could also convey useful information.

#### Dependence on stimulus: narrow vs. broadband

One intriguing argument is brought by the experimental work of Hermes et al., on human electrocorticography (ECoG) responses in V1/V2/V3 to a range of flashed visual stimuli (Hermes et al., 2015a). The authors make a distinction between narrow- and broad-band gamma oscillations that are likely produced by different substrates, as shown by other studies (Senzai et al., 2019; Sirota et al., 2008; Welle and Contreras, 2016). They show that narrow band gamma (30–80 Hz) is induced reliably only by gratings. Noise patterns, faces, and houses, induced narrowband gamma only in a fraction of the recorded electrodes (Hermes et al., 2015a). By contrast broadband gamma seems to be better represented across multiple sites and for all tested stimuli. They conclude that narrow-band gamma oscillations are not necessary for visual perception (Hermes et al., 2015b) and suggest that broadband gamma, reflecting asynchronous activity, can support information transmission and perception (Hermes et al., 2015a). Even if narrowband gamma is not always necessary for visual recognition it does not mean that gamma has no role in perception. Gamma has been shown to enhance tactile stimulus detection (Siegle et al., 2014) and to improve stimulus discrimination in silico (Schaefer et al., 2006). In monkey visual cortex it encodes stimulus spatial features, being induced when the receptive field can be predicted from the surrounding context, even if firing rates do not change (Peter et al., 2019).

Binding by synchrony (Singer, 1999) has been contested on grounds that visually induced gamma frequency is dependent on stimulus properties (Peter et al., 2019; Ray and Maunsell, 2015), especially on contrast (Henrie and Shapley, 2005; Ray and Maunsell, 2011; Roberts et al., 2013). According to this criticism, gamma is unfit to bind together parts with different contrasts into a coherent perception of a whole (Ray and Maunsell, 2015) because it is unclear how circuits with different frequencies would enable synchronization, or the maintenance of some stable phase relation for CTC and PC. Others have argued that this conundrum can be settled with a dynamic perspective, whereby oscillations of different frequencies can still be bound together by phase relations that systematically reflect the stimulus properties (Lowet et al., 2015). Information about the whole can be recovered from the phase, even if frequencies are different (Lowet et al., 2015).

#### Phase disruption due to broad-band contamination and conduction delays

Synaptic transmission between areas introduces delays on time scales comparable with the gamma cycle. In monkeys the lags of visual responses can span up to 70ms (Schmolesky et al., 1998). Within local V1 circuits the upper layer neurons fire with a delay of about 10–15ms compared to the input layer L4 (Best et al., 1986; Maunsell and Gibson, 1992). These delays depend on the distance between various areas and, because of their diversity and scale, are thought to impede precise synchronization within a single gamma cycle (Ray and Maunsell, 2015). Thus, given the unlikely presence of a central oscillator to synchronize sites, binding by synchrony, which requires zero-lag synchronization, may be applicable only in a small local neighborhood and not across areas. Importantly, this argument can be addressed by results showing synchronization of oscillators via dynamic relay elements. A central relay, like the thalamus, can help distant areas attain zero-lag synchronization (Gollo et al., 2010; Vicente et al., 2008), and this seems to be a universal mechanism, observed even in lasers (Fischer et al., 2006).

To conclude this section, the major problems of several studies that have investigated gamma oscillations are that they ignore the transient nature of gamma and they apply improper detection and quantification methodology. Indeed, in most cases TFRs (spectrograms) are averaged across trials for a certain stimulus and this can yield misleading results. Narrow-band gamma bursts spread in frequency in individual trials can mimic broadband gamma in the averaged spectrum. Also, an increase or decrease in gamma power in the averaged spectrum can be faked by, respectively, the systematic or unsystematic time-frequency localization of gamma bursts across trials, even if these bursts do not change in power. In addition, classical analysis techniques, based on the Fourier transform or single wavelets, have a tendency to smear out representations of higher frequency bursts and frequency leakage can also lead to the severe masking of the high-frequency bursts by lower frequency components (Moca et al., 2021). To correctly quantify the presence and properties of gamma oscillations, single trial analyses are required (Ardelean et al., 2023; Tal et al., 2020) and tools that can better localize brief oscillation bursts (Ardelean et al., 2023; Bârzan et al., 2022; Chandran Ks et al., 2018; Durka and Blinowska, 1995; Moca et al., 2021).

## Entrained gamma oscillations as a therapeutic tool

We have introduced the major terminological concepts related to gamma oscillations and have reviewed extensively their generation mechanisms and putative functional role in perception and cognition. However, a new direction has emerged in the past few years, where entrained gamma seems to be a useful therapeutic tool, prompted by its efficacy as a non-invasive treatment option for AD. We will review this new direction here, referring to concepts and mechanisms that have been described in the previous sections of the review.

### Relevance of gamma entrainment for the therapy of Alzheimer’s disease

AD is a slowly progressing neurodegenerative brain disorder that affects around 50 million people globally (Jagaran and Singh, 2021). Recent projections estimate that this figure is set to double every 20 years, potentially reaching 153 million patients by 2050. Its molecular pathophysiology is not yet fully understood, but one of the core hypotheses that has persisted for the last three decades is that accumulation of extracellular deposits of amyloid beta (Aβ) and intracellular neurofibrillary tangles of tau (i.e. amyloid hypothesis) interferes with neuronal circuitry (Busche and Konnerth, 2016), alters neuronal firing rates (Kellner et al., 2014), and disrupts the rhythmic activity within the gamma band (Chung et al., 2020), ultimately leading to neuronal death and severe cognitive dysfunction.

Despite significant advancements in AD diagnostics (Bucci et al., 2021; Hansson et al., 2018; Bucci et al., 2013) effective strategies to treat or reverse the disorder are missing. Currently, the best treatment protocols primarily focus on symptomatic management and can only delay the progression of the disorder. For example, one of the latest promising molecular treatments to tackle AD symptomatology is immunotherapy using monoclonal antibodies that target Aβ plaques, such as *aducanumab* (Rahman et al., 2023). Despite its efficacy in reducing Aβ buildup, several clinical trials have reported significant adverse effects, including brain hemorrhage and edema (Rahman et al., 2023). These side effects raise important safety issues regarding its clinical use, representing a potential step back in the AD care.

In response to the rapid increase and socio-economic burden of AD, scientists are branching out from traditional methods in exploring AD pathogenesis, approaching the disorder from different angles and perspectives to uncover potential treatments. Multiple studies have identified a fundamental link between abnormal gamma activity and AD (Byron et al., 2021; Goutagny and Krantic, 2013; van Deursen et al., 2008). A promising line of investigation and treatment therefore targets brain oscillations, particularly within the gamma band, as a new therapeutic hope in AD pathology.

Accumulating evidence indicates that gamma-generating mechanisms are impaired in AD pathology, resulting in altered levels of gamma oscillatory activity (Traikapi and Konstantinou, 2021), in both humans and animal models of AD. Moreover, in vivo studies conducted in the hippocampal area CA1 have established that a reduction of gamma activity results in cognitive dysfunction and memory impairment (Mably et al., 2017). It is therefore not surprising that gamma activity is pivotal in AD pathophysiology, with recent studies indicating that aberrant gamma oscillations may even be considered as an early biomarker of AD. In fact, Malby and Colgin have found that both aberrant gamma activity and cognitive dysfunction manifest prior to Aβ buildup (Mably et al., 2017; Mably and Colgin, 2018).

Several studies explored the use of multi-sensory stimulation at 40 hz and suggested that promoting gamma oscillations in the brain through neural entrainment constitutes a viable option as a non-invasive treatment to prevent and ameliorate AD symptomatology (Adaikkan and Tsai, 2020; Iaccarino et al., 2016; Martorell et al., 2019). An early study conducted by Iaccarino et al., 2016 entrained gamma activity through light flickering in an animal model of AD, and found that an exposure to a specific 40 hz flicker frequency in the regimen of 1 hr daily for 7 days reduced Aβ levels by approximately 50% and enhanced cognitive performance. This study, which introduced the GENUS therapy, has laid the groundwork for ongoing research, fueling interesting debates about sometimes conflicting results, but inspiring further recent developments.

Spanning nearly a decade, a multitude of studies have validated the benefits of GENUS in both animal models of AD and human patients. The concept of this novel therapy hinges on eliciting neural entrainment in the brain *via* sensory stimuli, such as auditory and/or visual. The frequencies of these stimuli can vary, but it is well documented that the specific frequency of 40 Hz, as opposed to 20 Hz or 80 Hz (Martorell et al., 2019), enhances gamma power across several brain areas which, in turn, promote microglial phagocytosis of Aβ (Martorell et al., 2019). An unavoidable question in this debate is why neural entrainment at precisely 40 Hz could offer significant advantages in combating Alzheimer’s disease pathology. Insights from older research may shed light on this.

First, the 40 Hz brain rhythm may be essential in both higher (i.e. feature binding) and lower brain function (i.e. phase coding of neuronal activity; Fries, 2005). Second, experimental evidence has shown that under tonic excitation, networks of mutually inhibitory interneurons in the hippocampus exhibit intrinsic oscillations and collectively ‘resonate’ at 40 Hz (Jefferys et al., 1996). This phenomenon, also referred to as the interneuronal network clock, plays a vital role in sensory information processing, tuning the frequency of the gamma rhythm across different brain areas (Jefferys et al., 1996). Third, gamma activity centered around 40 Hz is functionally relevant for memory-related core brain areas, such as hippocampus and neocortex (Headley and Weinberger, 2011; Jirakittayakorn and Wongsawat, 2017). Fourth, two seminal studies Cardin et al., 2009; Sohal et al., 2009 have demonstrated that fast-spiking, PV^+^ interneurons in cortex tune the cortical circuitry to a 40 Hz resonance operating regime. Given all these considerations, one can postulate that 40 Hz serves as an intrinsic natural rhythm essential for specific cognitive processes that become primarily dysfunctional in AD. The disruption of this rhythm may be a proxy for the dysfunction of cellular and molecular mechanisms associated with AD neuropathology.

Further evidence to support the importance of the 40 Hz entrainment is brought by studies investigating auditory steady-state responses (ASSRs) and their link to GABAergic transmission. Indeed, a study by Parciauskaite et al., 2019 on healthy young males has found that 40 Hz auditory stimulation is positively correlated with cognitive abilities such as planning and problem solving. Additionally, a study conducted by Huang et al., 2023 has found that 40 Hz visual stimulation enhances specific behaviors in adult C57BL/6J mice, including social exploration, olfactory abilities, and short-term memory. Enhancement of olfactory function after 40 hz light stimulation has also been confirmed in AD mice (Hu et al., 2024). Furthermore, 40 Hz light flickers ameliorates stress-related behaviors and inhibits neuroinflammation in chronically stressed mice (Yao et al., 2024). Finally, an interesting piece of evidence is brought by studies investigating synaptic plasticity, showing that specific neural entrainment at 40 Hz in wild type mice induces hippocampal neuroplasticity remodeling and promotes learning and memory via LTD (Tian et al., 2021).

### GENUS in animals

The first evidence of 40 Hz entrainment’s efficacy (as opposed to 20 Hz or 80 Hz) in reducing amyloid buildup in two distinct AD mouse models was reported by Iaccarino and colleagues in 2016. A few years later, a study conducted by Adaikkan et al., noted similar effects in two distinct animal models of neurodegeneration, specifically P301S and CK-p25. Moreover, this research found that gamma entrainment reached beyond visual regions to engage higher brain areas, including the hippocampus, somatosensory cortices, and prefrontal cortex (PFC; Adaikkan et al., 2019). This suggests an enhancement in functional connectivity throughout the brain, uniquely associated with 40 Hz stimulation (Adaikkan et al., 2019). Following the same premises, a follow-up study by Martorell et al., combined visual and auditory stimuli administered in the same regimen in two animal models of AD: 5XFAD and P301S. The results were promising: 40 Hz stimulation successfully reduced the Aβ buildup and tau-phosphorylation across multiple areas of the neocortex (Martorell et al., 2019). This reduction was not observed with administration of other stimulation frequencies (i.e. 8 Hz, 80 Hz) and the effect was more pronounced when both auditory and visual stimuli were used.

A recent study by Suk et al., explored the potential of 40 Hz stimulation through a distinct sensory modality, namely whole-body vibrotactile stimulation, as a novel and non-invasive therapeutic approach in treating neurodegenerative disorders associated with motor dysfunction. The study’s results indicated that, across two distinct neurodegeneration animal models, tau P3S01 and Ck-p25, regular exposure to 40 Hz vibrotactile stimulation for 1 hr daily over multiple weeks enhanced neural activity in crucial areas: the somatosensory cortex and the primary motor cortex. Additionally, in both animal models, a reduction in brain pathology was noted within these regions alongside an improved performance in motor tasks (Suk et al., 2023).

An additional attempt to replicate the initial findings by Iaccarino et al. was undertaken by Bobola et al. in 2020. In this study, 5XFAD mice, aged between 2 and 4 months, were exposed to a transcranially focused ultrasound (tFUS) pulsed at 40 Hz, either acute or chronically. The results confirmed that chronic administration over 5 days of 40 Hz tFUS reduced the Aβ buildup by 50% compared to the control (sham) group. Furthermore, 1 hr acute administration of 40 Hz tFUS increased microglial activation around Aβ buildup more so than in the control (sham) group or untreated hemisphere (Bobola et al., 2020).

Additional investigations employing optogenetic techniques have also explored the therapeutic potential of 40 Hz stimulation. One study conducted by Etter et al., delivered different frequency pulses in order to activate medial septal PV^+^ interneurons in the hippocampus of the J20-APP mouse model, known for its spatial memory impairment and decreased lower gamma amplitude. The authors found that stimulation at 40 Hz, and not other frequencies, was able to rescue hippocampal slow gamma oscillations. Additionally, within the same study, 40 Hz stimulation enhanced spatial memory during retrieval despite significant amyloid deposits (Etter et al., 2019).

Another study by Wilson et al., 2020, has found contradictory results. The authors used the 5xFAD animal model and reported that optogenetic activation of PV^+^ interneurons in the basal forebrain led to an increase in Aβ deposits, rather than reducing them as observed in previous studies (Wilson et al., 2020).

### GENUS in humans

Pilot human studies of non-invasive stimulation techniques such as conventional GENUS (40 Hz light and/or sound signals) conducted on human subjects highlighted the tolerability, adherence, and safety of flicker therapy in human participants, with high rates of tolerability and adherence observed over 4–8 weeks of treatment. Adverse events associated with flicker treatment were mostly mild, indicating a favorable safety profile (Chan et al., 2022; He et al., 2021). Furthermore, these studies have revealed alterations in cytokines and immune factors in the cerebrospinal fluid (He et al., 2021), increased functional connectivity in the default mode network (Chan et al., 2021; He et al., 2021), reduced ventricular dilatation and improvements in cognitive functioning and circadian rhythmicity (Chan et al., 2021).

Additional non-invasive modalities for brain stimulation include tACS and TMS. Emerging evidence indicates that 40Hz-tACS delivered in the PFC of healthy subjects improves their cognitive performance by reducing the response latencies in solving complex logic problems (Santarnecchi et al., 2016). Another study, involving two 30 min tACS sessions/day combined with cognitive exercises in both the active and sham (cognitive exercises, no tACS) groups, showed memory improvement, with the active group exhibiting significantly better retention of memory after one month compared to the sham group (Kehler et al., 2020). Similarly, another study reported memory enhancement and restoration of intracortical connectivity supporting cholinergic neurotransmission in comparison to sham therapy (Benussi et al., 2021). These findings collectively highlight the effectiveness of 40 Hz rhythmic stimulation in enhancing memory and neurological connectivity, underscoring their potential as therapeutic strategies for cognitive enhancement.

Despite numerous studies demonstrating a significant decrease in amyloid load following gamma entrainment at 40 Hz in mice models (Iaccarino et al., 2016; Lee et al., 2018; Martorell et al., 2019) the applicability of these findings to human subjects has not been fully established as current evidence does not indicate a notable reduction in amyloid load in humans (Dhaynaut et al., 2022; Ismail et al., 2018). The study conducted by Ismail et al. suggests that the reason for this discrepancy could be that the duration of the treatment in human trials might not have been long enough to produce noticeable changes.

Transcranial magnetic stimulation (TMS) is explored as a non-invasive depression treatment, offering improved tolerability compared to electroconvulsive therapy (ECT). Studies reveal reduced TMS-evoked potentials in AD patients and diminished EEG alpha frequency reactivity in amyloid-positive individuals without dementia, suggesting potential applications in early AD detection and intervention (Chan et al., 2021). Moreover, recent findings demonstrate cognitive function improvement, enhancement of gamma band power in the left temporoparietal cortex, as well as increased local, long distance, and dynamic connectivity within the brain (Liu et al., 2022).

Invasive stimulation techniques, such as deep brain stimulation (DBS), have become established in the treatment of Parkinson’s disease and essential tremors, but are only now becoming possible candidates for a broader range of diseases (Chan et al., 2021). For instance, a novel therapeutic avenue for AD, based on DBS, involves bilaterally stimulating the fornix with short, high-intensity (>3 V) pulses at a frequency of 130 Hz (Lozano et al., 2016), while another study reports improvement of treatment-resistant major depression using DBS with a frequency of 100 Hz (Scangos et al., 2021). This last study also leads nicely into the final category of techniques, namely closed-loop stimulation, as authors of that study also employed an algorithm that identifies subject-specific biomarkers of depression and delivers DBS as necessary.

Technically, closed-loop stimulation is relatively straightforward: use a control signal, for example gamma oscillatory activity, to control the parameters of stimulation, for example the frequency and intensity of light flicker stimulation. Closed-loop systems are very popular with direct stimulation (tACS, rTMS, DBS) and are under investigation for the treatment of a myriad of conditions (Sellers et al., 2024). However, on the (external) sensory stimulation front, it appears that research is scarce on closed-loop stimulation, such that it remains an open avenue for research.

### GENUS and cognitive performance

While in previous sections we focused on the physiological evidence for gamma entrainment as a potential treatment for Alzheimer’s, such as the reduction in the presence of Aβ, here we will focus on studies showing increase in cognitive performance evaluated *via* a behavioral proxy. Martorell et al., 2019 found that multimodal GENUS increased memory performance on the novel object recognition task as well as on the novel object location task in the 5XFAD mouse model of Alzheimer’s. Park et al., 2020 showed recovered memory performance on step through and Morris water maze tasks in an Alzheimer’s mouse model when exposed to concomitant 40 Hz stimulation and exercise. These effects were also associated with a host of biological measurements in the hippocampus. Kim et al., 2024 similarly showed a recovery from cognitive deficits through GENUS treatment in a mouse model of chemotherapy. You et al., 2020 found improved reaction times in an attention task in humans when applying 40 Hz flicker, but not for lower frequencies. Khachatryan et al., 2022 showed that by administering GENUS during cognitive tasks, they could increase the power entrainment effect, but did not measure performance on the cognitive tasks. These results provide indirect evidence for the role of gamma oscillations in cognition by showing that we can recover or improve performance on cognitive tasks by entraining them.

### GENUS and sleep

A recent study by Zhou et al., 2024 has also investigated the somnogenic effect of 40 hz light flicker stimulation. Sleep disruptions are common in several neurodegenerative disorders (Raggi and Ferri, 2010) and some studies suggest that disturbances of sleep may precede the onset of symptoms in such disorders, potentially serving as a precursor of disease manifestation (Musiek and Holtzman, 2016). The exact mechanisms through which sleep might play a role in these disorders still need to be elucidated. However, (Zhou et al., 2024) reported that 40 Hz light flicker stimulation enhances extracellular adenosine levels through ENT2 signaling, leading to a somnogenic effect in different animal models and improvement of sleep quality in children. The same study also identified that the cellular source of adenosine stems from neuronal interactions of both excitatory and inhibitory neurons, rather than astrocytes, mirroring the same mechanism that gives rise to gamma oscillations. The integration of these new findings with the previous research (Murdock et al., 2024) links the glymphatic system with sleep homeostasis (Bozzelli and Tsai, 2024) in response to 40 Hz stimulation. It has been shown that sleep is an important factor in AD (Cordone et al., 2019) through its role in clearing the brain of toxic metabolic by-products, including amyloid plaques and tau tangles. We also know that gamma oscillations appear during REM and delta sleep (Llinás and Ribary, 1993). While most of the clearing has been proposed to happen during slow wave sleep (where gamma is normally absent; Cordone et al., 2019) there may still be a link between these two processes.

### Contrastive views on GENUS

Visually-entrained gamma has been reported by a battery of studies in the primary sensory cortex in anesthetized, head-restrained, and freely moving mice (Adaikkan et al., 2019; Iaccarino et al., 2016; Martorell et al., 2019). However, what remains debated is the entrainment of the oscillatory activity in other areas outside the primary sensory cortex.

Reports of visual entrainment of 40 Hz oscillation in PFC or areas of the hippocampus alongside sensory areas support the propagation scenario (Adaikkan et al., 2019; Iaccarino et al., 2016; Martorell et al., 2019). Also, human EEG studies have reported small amplitude gamma entrainment in parietal and frontal regions (Herrmann, 2001; Jones et al., 2019; Pastor et al., 2003). Auditory stimulation alone does not lead to a significant increase in LFP power at 40 Hz in CA1 and medial prefrontal cortex (mPFC). However, combined auditory and visual periodic stimulation causes LFP power to increase at 40 Hz in CA1, with a very small increase in mPFC (Martorell et al., 2019).

A second level of this debate postulates that even if oscillatory activity entrained by sensory stimulation reaches deep areas, such as the hippocampus, or higher areas such as the PFC, this does not translate in a significant effect on the pathological markers of Alzheimer’s disease. One variable, which contrasts between studies, is the pathology marker targeted, which is strictly coupled with the genetic model used in the studies. Initially, Iaccarino et al. looked at 5XFAD and tau P301S mice and showed decreased Aβ levels, plaque load and mutant tau levels in primary visual areas, but no effects in the hippocampus or barrel cortex (Iaccarino et al., 2016). Later on, Adaikkan et. al. evaluated the effect of sensory stimulation on tau P301S mice and confirmed the lack of effect on mutant tau levels, but revealed a significant effect on neuronal loss in both V1 and CA1. Additionally, 40 Hz flicker stimulation in severe neurodegeneration model CK-p25 mice was associated to a reduction in pathological markers, such as brain mass loss, cortical shrinkage, ventricular expansion, and neuronal loss in V1, CA1, and SS1 (Adaikkan et al., 2019).

The different nature of these pathology markers suggests on one hand that gamma has a potential to ‘turn back the clock’ on the progression of incipient stages of neurodegenerative diseases, such as Alzheimer’s. On the other hand, reducing neuronal loss when the disease progression has reached more advanced stages aims to delay or halt the symptomatology cascade, rather than to reverse pathology. Given the wide variety of disease models in which positive effects have been observed in relation to sensory periodic stimulation, it seems like the mechanism of action is not specific to molecular particularities of disease, but rather a more generic mechanism such as neuronal circuitry maintenance, as proposed by Tsai et al.

Another factor is the duration of the visual flicker protocol application. Initially, Iaccarino et. al. started with 1 hr per day, 7 days of protocol application, and Martorell et al. used the same duration for auditory and multi-sensory stimulation. Adaikkan increased protocol application to up to 6 weeks for the neuronal loss study in order to see significant results.

A third factor which seems to influence the visibility of therapeutic effects of periodic stimulation is the sensory modality: Initially, studies relied heavily on visual flicker stimulation (Adaikkan et al., 2019; Iaccarino et al., 2016). Surprisingly, auditory periodic stimulation has proved to be more efficient and reliable in generating effects on pathological markers of Alzheimer’s disease, even if it seems to be less effective in LFP gamma entrainment in CA1 (Martorell et al., 2019). In fact, in 5XFAD, APP/PS1, and tau P301S mice, an auditory protocol application duration of 7 days is sufficient to cause significant Aβ plaque load and mutant tau decrease in the auditory cortex and hippocampal areas. Applying visual and auditory periodic stimulation concomitantly spreads this beneficial effect throughout higher areas of the neocortex.

Contesting these views, Soula et al., 2023 reported results showing that 40 Hz light stimulation does not engage native gamma oscillations in the hippocampus or the visual cortex and therefore raised doubts about the possibility of using this as a therapeutic strategy in neurodegenerative diseases (Soula et al., 2023). Specifically, the study reports a much lower decrease in Aβ levels in V1 and hippocampus than previously reported, in both acute and chronic 7-day protocol application. This has been contested later by Carstensen et al., suggesting that a higher sample size and more accurate statistical tests would increase the significance of the reported effects (Carstensen et al., 2023).

However, the arguments brought forward by Soula et al. are notable and might spring potential research directions to improving the effectiveness and reproducibility of therapeutic effects of GENUS and towards a more complete understanding of the underlying mechanisms of these effects. First, they argue that the oscillatory component observed when applying the 40 Hz visual stimulation does not consist of a change in native gamma power increase, but of a steady-state, narrow band 40 Hz oscillatory response in V1, which does not propagate to deeper or higher areas. They propose lower frequencies, like 4 Hz, as better candidates for highly efficient, wide-spread propagation of oscillatory activity. Additionally, they argue that 40 Hz stimulation without behavioral saliency leads to the habituation of neurons in the hippocampus and therefore results in the failure of propagation of the V1 steady-state response to the hippocampal structures. This argument leads to the intuition that it might be useful to integrate a more specific task in the stimulation paradigm and techniques such as neurofeedback, in order to increase the therapeutic outcome of the method.

And third, they report that 40 Hz stimulation is aversive for mice and might cause eyestrain and fatigue. Stimulation at close frequencies (30–60 Hz) have been reported to enhance acetylcholine levels in the hippocampus (Soula et al., 2023), reduce perineuronal nets (Venturino et al., 2021), and even increase mutant tau levels (Wu et al., 2016). They conclude that gamma band frequency stimulation should be used with caution, as it has the potential to both decrease and increase pathology markers, depending on the particular circumstances. This raises the importance of identifying a real-time indicator of treatment outcome, and a flexible stimulation control loop, with potential resting periods being instrumental for the positive outcome of such treatments.

## Gamma’s hidden role: Balancing the brain’s internal ecosystem

We have reviewed the major mechanisms of gamma oscillations and discussed two main reasons it is important: gamma’s role for brain function and gamma as a therapeutic tool. Gamma’s role has been extensively studied in the context of perception, cognition, and behavior. A picture emerges where it seems to be instrumental for the encoding and transmission of neural information across brain structures, as well as for coordination of circuit activity. Substantial critique also exists, but advancements in quantification methodology as well as design of causal experiments will hold the key to resolve once and for all the question regarding its utility for brain function. On the other hand, a novel field has recently emerged where gamma is studied for its therapeutic benefits, especially for the treatment of AD. Controversy is no stranger to this field either: It is debated whether stimulation using gamma frequencies propagates to large enough territories and if this propagation has the expected therapeutic effect in AD.

The recent studies by Tsai and others have made significant strides, revealing that the entrainment of brain circuits into gamma oscillations at 40 Hz seems to impact microglia, astrocytes, and the glymphatic system, as pivotal protagonists orchestrating the removal of brain metabolic waste by facilitating the movement of cerebral fluids (Murdock et al., 2024). The cerebrospinal fluid (CSF) enters the brain through perivascular spaces surrounding arteries, traverses the astrocyte endfoot layer, and flows through the parenchyma, gathering metabolic waste that is eventually drained along the veins (Bojarskaite et al., 2024). This is known as the glymphatic clearance pathway. Its efficiency in removing interstitial metabolic waste products, such as Aβ and tau, and relies mainly on astrocytic aquaporin-4 (AQP4) water channels (Bojarskaite et al., 2024). Clinical studies on AD patients have shown impairments in this system, suggesting that interventions targeting this pathway could be a viable treatment option for AD. The key findings can be summarized as follows. One hour exposure to 40 Hz multisensory audio-visual stimulation increased 40 Hz local field potentials in the PFC of 6 months-old 5xFAD mice and significantly reduced the amyloid buildup compared to no stimulation, 8 Hz, or 80 Hz (Murdock et al., 2024). The observed results are thought to occur because GENUS enhances arterial pulsatility (Murdock et al., 2024), which in turn regulates CSF dynamics. GENUS promotes the perivascular CSF influx and interstitial cerebral fluid (ICF) efflux by increasing polarization of aquaporin-4 water channels along the astrocytic endfeet. In the same study, it has been shown that this pathway is additionally vasoactive intestinal peptide (VIP) expressing interneuron-dependent: Arterial pulsatility is modulated principally by VIP^+^ interneurons through the frequency-dependent release of neuropeptides. These neuropeptides are thought to initiate the glymphatic clearance, which ultimately attenuates AD pathology (Murdock et al., 2024). This mechanism has been discovered in the context of GENUS stimulation, which is a powerful but unnatural sensory drive.

It could be that gamma oscillations generated endogenously by the brain during naturalistic perception, cognition, or sleep, may activate similar mechanisms. Therefore, inspired by, and as an extension of GENUS, here we propose an additional direction to explore. We hypothesize that endogenous gamma oscillations act as a ‘service rhythm’ that homeostatically maintains healthy brain function via a cascade of neural, glial, and vascular mechanisms. We suggest that a complex, yet little understood interaction between interneuronal circuits, involving multiple interneuron classes, takes advantage of gamma rhythmicity to regulate blood flow and clear metabolic waste products, preventing deposits and maintaining healthy circuits. This mechanism, which we call GAMER (GAmma MEdiated ciRcuit maintenance) is activated by endogenously generated gamma oscillations both in response to naturalistic stimuli and produced internally during memory recall, cognitive processing, or sleep. A breakdown or inefficiency of GAMER may promote neural circuit dysfunction and neurodegeneration, thus establishing a causal role for gamma. The nature of gamma oscillations that support GAMER is yet unknown and may be quite different from those entrained using GENUS. We will next discuss this new hypothesis and propose key experiments to test it.

GENUS relies on very strong periodic stimulation delivered using sensory inputs. However, in naturalistic settings, where input is received from active visual sampling (Gal et al., 2024), or via auditory or tactile modalities, a strong periodic component is usually not present. This does not mean that naturalistic input does not induce or evoke gamma oscillations, but their nature is expected to be different from those entrained by GENUS. Indeed, a recent study (Duecker et al., 2021) has investigated the relationship between gamma entrained by rhythmic photic stimulation and endogenously generated gamma using simultaneous visual stimulation with drifting gratings. The two processes give rise to distinct oscillations that do not interfere, suggesting that the nature of GENUS-entrained and endogenously generated gamma is different.

Endogenous gamma induced by non-periodic sensory inputs has been well documented in visual (Bartoli et al., 2019; Brunet et al., 2015; Chen and Farivar, 2020; Gray and Singer, 1989; Hermes et al., 2015b; Shirhatti et al., 2022), auditory (Gourévitch et al., 2020; Karthik et al., 2021; Lakatos et al., 2007; Steinmann et al., 2014), somatosensory (Cheng et al., 2016; Gross et al., 2007; Iwamoto et al., 2021; Tu et al., 2016), or olfactory (Yang et al., 2022) modalities. In addition, internally generated gamma oscillations, or a stronger synchronization of these oscillations across the cortex, have been observed during multiple high-effort cognitive tasks, such as meditation (Fell et al., 2010; Lee et al., 2018; Lutz et al., 2004; Martinez Vivot et al., 2020), motor imagery (de Lange et al., 2008; Gwon and Ahn, 2021; Smith et al., 2014), mental rotation (Bhattacharya et al., 2001; Nikolaev and Anokhin, 1998; Nishimura et al., 2020), or neurofeedback training in brain-machine interfaces (Engelhard et al., 2013; Merkel et al., 2018). A further study by Kawasaki & Watanabe found strong gamma band activity elicited by mental manipulations of color, shape, direction, and speed features of an object, highlighting the diversity of situations in which these oscillations appear (Kawasaki and Watanabe, 2007).

**Figure 3.**
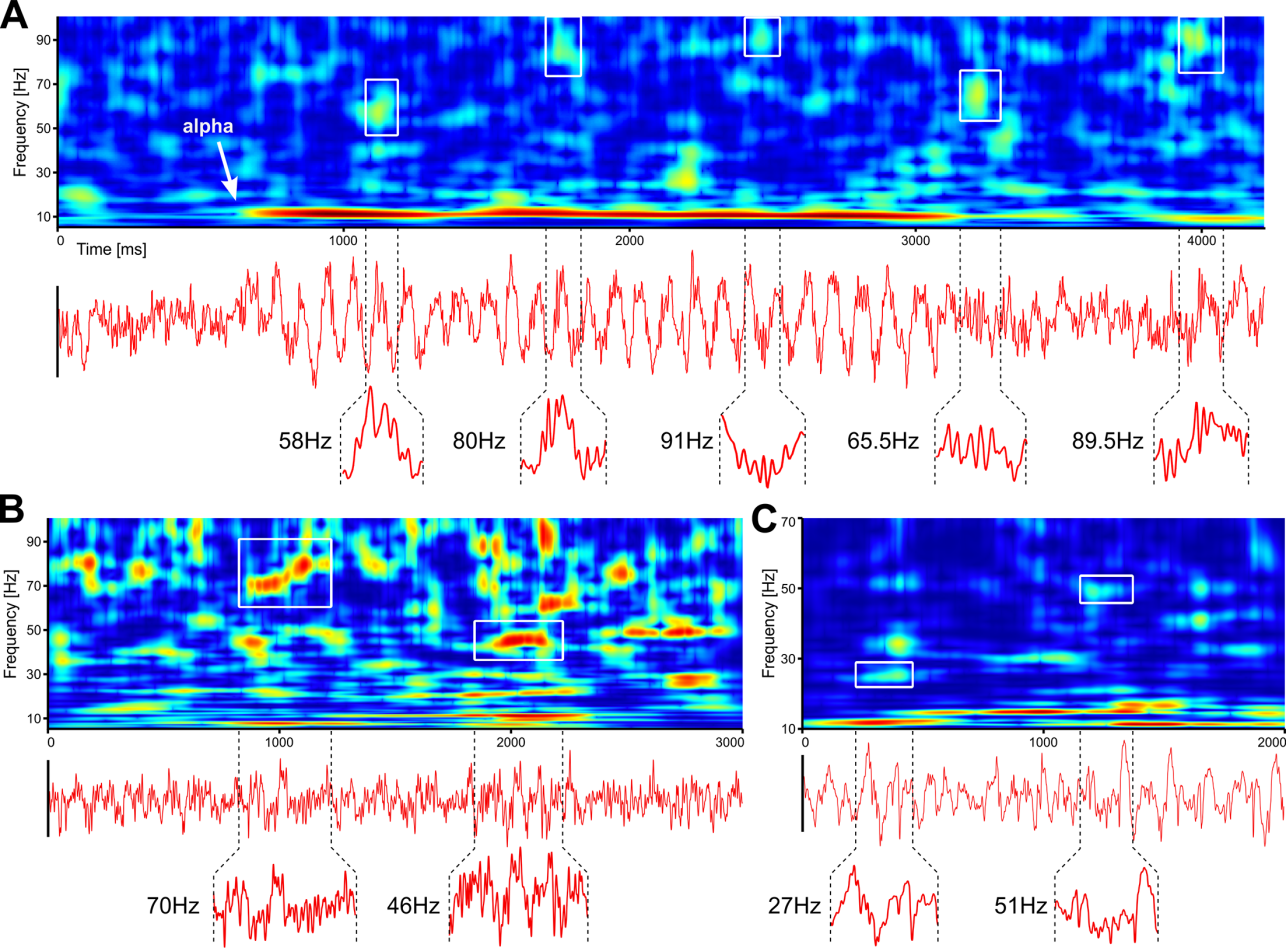
Robust gamma bursting in spontaneous activity recorded in humans and mice. (A) Resting state EEG (Oz electrode) recorded with eyes-closed in a human participant. (B) Same as in A, but on LFP recorded from an awake mouse. (C) Same as in B, but with spontaneous data recorded in an anesthetized mouse. Insets show time traces corresponding to gamma bursts outlined in the corresponding TFRs. TFRs were computed using superlets (Moca et al., 2021).

While frequently overlooked, spontaneous activity is also a major source of endogenous gamma bursting. In Figure 3, we show single trial analyses of resting-state human EEG and spontaneous LFP in the mouse. First, we show an example of human EEG where the subject closes the eyes (Figure 3A). This is immediately followed by the development of prominent alpha waves, which are accompanied by genuine, narrow-band gamma bursting over occipital electrodes. Thus, even in the absence of visual input, powerful gamma bursting can be found in EEG data. Second, Figure 3B and C show examples of gamma bursting in spontaneous activity recorded in awake and anesthetized mice, respectively. While gamma bursting is more prominent in awake animals, it can also be found under anaesthesia. We argue that gamma oscillations may be much more prevalent than currently thought and novel studies are required, using proper tools, to get a clearer picture of their expression under different brain states.

Sleep also promotes the development of endogenous gamma oscillations. For example, gamma bursting, mediated by fast-spiking interneurons, has been found to be tightly aligned to sleep spindles in both animals (Averkin et al., 2016) and humans (Ayoub et al., 2012). Takeuci et al. have observed cross-frequency coupling between high-frequency and slow wave oscillations during slow-wave sleep and a significant increase of gamma bursting during REM sleep in the hippocampus of unanesthetized primates (Takeuchi et al., 2015). Similarly, marked gamma oscillations are reported in humans during slow-wave (Le Van Quyen et al., 2010) and REM sleep (McKenna et al., 2017).

### The cortical interneuron circuitry as a relay for vasoactive regulation

**Figure 4.**
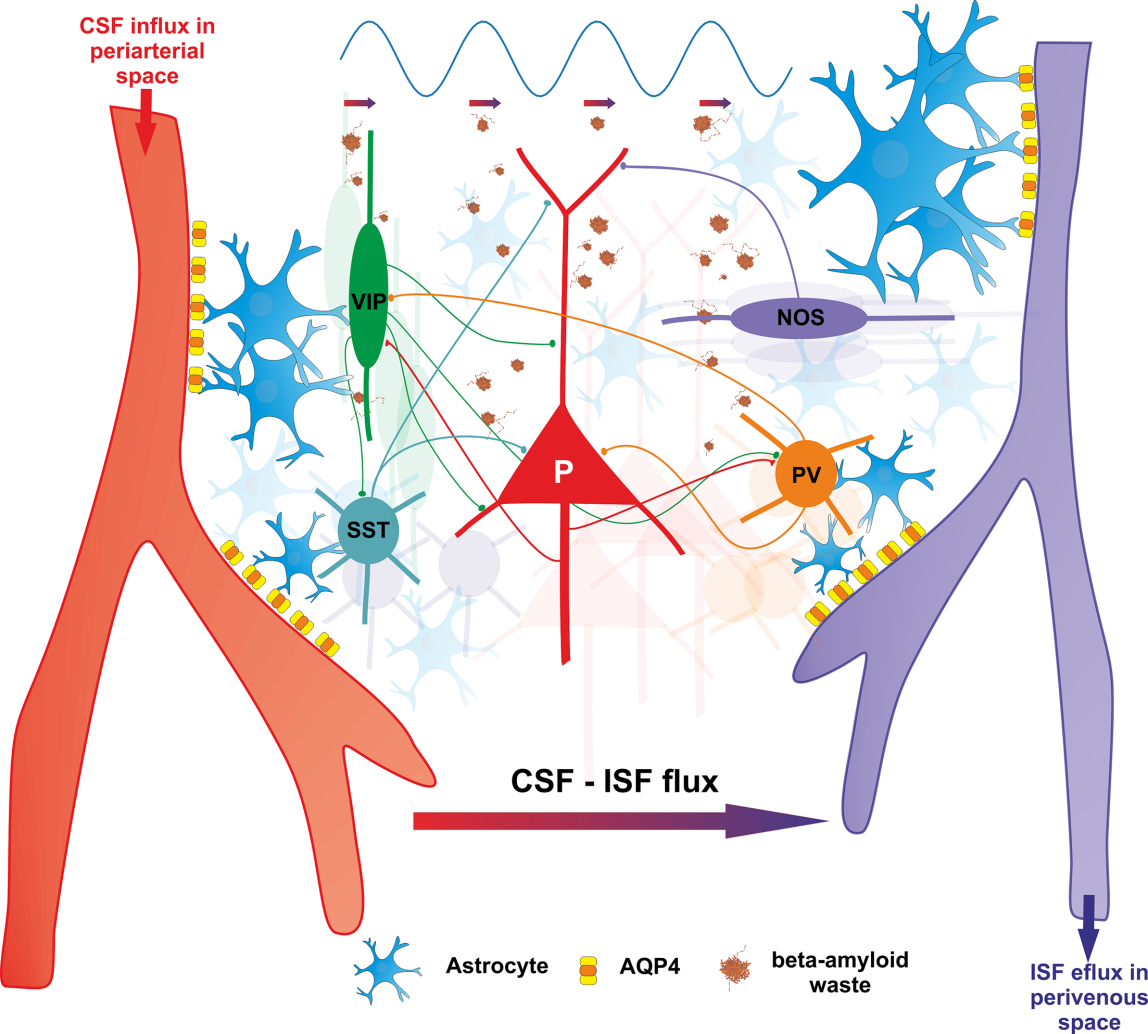
Vasomotor control by four major classes of interneurons in cortex (PV^+^, VIP^+^, SST^+^, NOS^+^). These interneurons, with vasomotor properties, are known to act as local integrators promoting neurovascular coupling and enhancing arterial pulsatility – essential for cerebral autoregulation. VIP^+^ interneurons enhance arterial pulsatility through release of frequency-dependent neuropeptides, promoting vasodilation. Conversely, SST^+^ interneurons promote vasoconstriction, while NOS^+^ and PV^+^ cells exhibit both vasodilatory and vasoconstrictive properties. The interplay between these four classes of interneurons, along with their interactions with principal cells, support gamma rhythmogenesis which in turn, activates the glymphatic clearance. Gamma rhythmicity is essential for circuit maintenance and efficient waste removal through CSF-ISF exchange, contributing to homeostatic regulation.

Endogenous gamma oscillations are ubiquitous in brain dynamics, in both awake and sleep states. The implication of the interneuronal machinery in the rhythmogenesis of endogenous gamma is well known. However, most of the focus so far has been on the contribution of the fast spiking PV^+^ cells (Cardin et al., 2009; Kim et al., 2016; Sohal et al., 2009) and much less is known about the involvement of other types of interneurons. In addition to PV^+^, VIP^+^, somatostatin (SST), and nitric oxide synthase (NOS) expressing interneurons may also be instrumental for the generation and propagation of gamma oscillations. Importantly, all these populations of interneurons have vasoactive properties (Figure 4), modulating blood flow and tissue oxygenation (Aksenov et al., 2022). While VIP^+^ interneuron activation has vasodilation effects, SST^+^ interneurons promote vasoconstriction (Cauli et al., 2004), and NOS^+^ may feature both (Aksenov et al., 2022). Their coordinated firing is likely to have critical effects on regulating blood flow and related neurometabolic processes in the brain (Krawchuk et al., 2020).

SST^+^ interneurons express somatostatin, which is a peptide hormone acting as an endogenous inhibitory regulator of neuronal functions (liguz-lecznar and Dobrzanski, 2022). Somatostatin causes vasoconstriction and increases vascular permeability in the nervous system (Long et al., 1992). SST^+^ interneurons are a major class of inhibitory neurons in the cortex, mainly exerting dendritic inhibition (Fino et al., 2013). Not surprisingly, their dysfunction has been associated with several brain disorders, including AD (Song et al., 2021). The implication of SST^+^ in gamma oscillations is gradually beginning to be recognized. Hakim et al., 2018 have observed narrow-band 30 Hz oscillations in acute brain slices of mouse visual cortex in response to patterned optogenetic stimulation. Such oscillations were supported by an interplay of L2/3 pyramidal principal cells and SST^+^ interneurons, but did not involve PV^+^ cells, and appeared to be coherent across large distances. Antonoudiou et al. have found that SST^+^ and PV^+^ interneurons contribute together to gamma rhythmogenesis in the hippocampus (Antonoudiou et al., 2020).

Another important class of interneurons is the VIP-expressing class, which inhibits SST^+^ interneurons (Krabbe et al., 2019), having a disinhibitory effect on principal cortical cells (Millman et al., 2020). The vasoactive intestinal peptide promotes vasodilation, being a major regulator of cerebral blood flow (Cauli et al., 2004). It was recently suggested that VIP^+^ interneurons modulate cortical activity and sensory context-dependent perceptual performance (Ferguson et al., 2023). They also enhance responses to weak but specific stimuli (Millman et al., 2020). While it is not yet clear how VIP^+^ interneurons are involved in gamma rhythmogenesis, it has been shown that VIP^+^ interneurons in mouse cortex scale gamma power in a linear way, without changing its selectivity to the stimulus, and also suppress synchronization at larger distances, when different regions process non-matched stimuli (Veit et al., 2023). VIP^+^ interneurons could therefore tune gamma coherence across larger cortical territories (Veit et al., 2023).

Two studies have examined closely the importance of VIP^+^, PV^+^, SST^+^, and NOS^+^ interneurons for vascular control. Cauli et al., 2004 performed patch-clamp recordings in rat cortical slices with concomitant confocal imaging of biocytin-filled neurons, and laminin-stained microvessels. They have found that firing of interneurons was accompanied by a dilation or constriction of neighboring microvessels, with a predominantly dilatatory effect for VIP^+^ and NOS^+^, and constrictive effect for SST^+^, thus transmuting neuronal signals into vascular responses. In another study, employing optogenetic stimulation of VIP^+^, PV^+^, SST^+^, and NOS^+^ inhibitory interneurons, it was found that NOS^+^ stimulation promotes an increase in cerebral blood flow (CBF), SST^+^ stimulation is followed by a non-monotonic response profile, with initial increase and then decrease in CBF, VIP^+^ did not yield detectable changes in CBF, while slower increases in CBF were observed during PV^+^ entrainment (Krawchuk et al., 2020). Because they are reciprocally coupled (Ferguson et al., 2023; Lee et al., 2013) and have vasomotor properties (Cauli et al., 2004), it becomes clear that the coordinated firing of these interneuron populations is crucial in controlling not only cortical circuit dynamics but also blood flow. The challenge of GAMER is to determine how endogenous gamma oscillations affect the firing of these populations in vivo.

### Is 40Hz necessary?

Experiments with GENUS have demonstrated that specific 40 Hz stimulation is required in order to observe AD-related effects on cortical circuits, and not other frequencies, like 80 Hz (Murdock et al., 2024). We argue that while this frequency may be essential for GENUS, it may not be required when it comes to endogenous gamma (GAMER). In fact, 40 Hz stimulation is probably important for GENUS because it can leverage circuit resonance (Cardin et al., 2009; Sohal et al., 2009) and obtain efficient propagation across larger cortical territories. However, in vivo endogenous oscillations, generated across a large number of internal sources (Fernandez-Ruiz et al., 2023) could be synchronized via thalamic relay elements (Gollo et al., 2010; Vicente et al., 2008) and would not necessarily rely on circuit resonance, possibly benefiting from other oscillations frequencies.

The critical question here is whether the VIP^+^, PV^+^, SST^+^, and NOS^+^ interneuron complex behaves in a special way when engaged into oscillations at 40 Hz versus other frequencies. We do not have a clear answer at this point. It is known that gamma oscillations span a large frequency range, with the observation that, when one examines the PSD, the gamma band seems to be functionally split into high (>60/80 Hz) and low frequency (<60/80 Hz) sub-bands (Ray and Maunsell, 2011). It is still unclear if the two distinct gamma ranges share the same gamma generation mechanisms, but it is likely that both sub-bands are supported by engaging the interneuron populations, albeit under different conditions.

Differential engagement of interneuron populations has been shown before. For example, Antonoudiou et al. showed that SST^+^ and PV^+^ interneurons contribute differentially to regulating gamma frequency and that their interplay is far from linear (Antonoudiou et al., 2020). High (>60 Hz) and low (<60 Hz) frequency gamma oscillations are generated depending on the relative activation of the excitatory-SST and excitatory-PV loops. A recent study showed that both SST^+^ and PV^+^ neurons are involved in visually induced gamma oscillations, but with a differential delay relative to principal cell firing, the PV^+^ cells firing earlier in the gamma cycle (Onorato et al., 2023).

Another argument for transcending 40 Hz stimulation used in GENUS originates in observations on visual perception. Several studies have shown that certain types of visual stimuli, like superimposed (Wang et al., 2021) or large gratings (Murty et al., 2018) induce more than one gamma peak, indicating the presence of multiple gamma generators. In one study, three distinct generators were identified as a function of the spatial frequency of the stimulus (Han et al., 2021). While for GENUS, entrainment might be critically linked to the 40 Hz circuit resonance, GAMER could benefit from the endogenous oscillations produced by multiple gamma generators.

To conclude, other gamma frequencies than 40 Hz may be functionally useful in the GAMER hypothesis. However, we cannot exclude that different frequency-specific effects are found for different interneuron classes.

### Effectiveness of GAMER

GENUS stimulation entrains a sustained gamma response in neural circuits, which, at least in laboratory settings, has two important properties: it is sustained across relatively long periods of time (compared to the period of gamma) and it evokes strong responses, at least in primary sensory cortices. As a result, the glymphatic clearance system could take advantage of robust and sustained rhythmic vasomotor modulation. By contrast, GAMER relies on endogenous gamma, which occurs mostly in brief bursts spread across a wide frequency domain. Future studies should elucidate at least two major issues that may prove critical in determining if GAMER is actually an effective mechanism, similar to GENUS. First, the prevalence of endogenous gamma bursting needs to be established, both for spontaneous activity and with sensory stimulation. It is the opinion of the authors that single trial analyses, coupled with appropriate estimation methods (Moca et al., 2021), will demonstrate gamma bursting to be a ubiquitous phenomenon. Second, causal experiments should be designed to determine to what degree gamma bursting, which occurs in short packets, can activate the glymphatic clearance described above. Bursting at diverse frequencies instead of a sustained entrainment at a single frequency may activate vasomotor mechanisms that are yet unknown. This should also be considered given the diversity of the interneuronal machinery (Soltesz, 2006).

While GENUS relies mainly on 40 Hz stimulation, because this frequency can propagate across cortical circuits due to their resonant properties (Cardin et al., 2009; Sohal et al., 2009), a similar requirement may not be needed for endogenous gamma under GAMER. Stochastic, endogenous bursting could instead propagate across cortical territories in a similar manner to avalanches (Beggs and Plenz, 2003). In fact, neuronal avalanches may be tightly related to endogenous gamma bursting events (Gireesh and Plenz, 2008; Miller et al., 2019).

### Proposal for causal experiments

The key prediction of GAMER is that endogenous gamma oscillations in the VIP^+^, PV^+^, SST^+^, and NOS^+^ interneuron complex are essential for homeostatic regulation of blood flow, oxygenation, and glymphatic clearance, promoting healthy neurometabolic function. It is known that these interneurons are essential for normal brain function. For example, a causal role has been proposed for the degeneration of SST^+^ interneurons in the development of AD (Song et al., 2021), because patients exhibit low SST^+^ expression in cortex and hippocampus (Davies et al., 1980). But is gamma activity in the interneuronal complex also necessary? What benefit would it bring? As proposed by Tsai et al. (Murdock et al., 2024), one possibility is that rhythmic vasomotor modulation enables glymphatic clearance involving, among others, aquaporin-4 (AQP4) water channels (Bojarskaite et al., 2024). Another possibility is that rhythmic vasomotor modulation may actively correlate the blood flow volume with the metabolic demands of the local circuit. In this scenario, rhythmicity is essential in order to ensure sufficient metabolic and oxygen supply because of its pumping effect on the microvessels. Indeed, it was shown a while ago that hemodynamic signals are correlated with cortical gamma (Niessing et al., 2005).

To test these scenarios, we propose a few critical experiments. First, one could simultaneously observe microvessels surrounding interneurons and use optogenetics to activate the latter using light pulses with either rhythmic patterning (gamma) or random inter-pulse intervals, while matching firing rates in both conditions. This would allow testing the effect of rhythmic versus random stimulation, while ruling out the contribution of the firing rate of the interneurons. Second, in a more complex experiment that should be performed in vivo, one could implant rodents with a chronic optogenetic stimulator that would be used to gently perturb a local population of VIP^+^, PV^+^, SST^+^, or NOS^+^ interneurons. The perturbation should kick interneuron firing out of phase, preventing its alignment to the endogenous gamma of the larger circuit. This second experiment would necessarily be a closed-loop one, where one can both observe the endogenous gamma of the larger circuit and use perturbation to break down the rhythmicity of the target interneuron population. Applying this protocol for several weeks, would then enable one to assess the impact of the perturbation on the local patch of cortex where it was applied and to contrast it with control analyses on cortical circuits where perturbation has not been applied, in the same animal. Importantly, as in the first experiment, the optogenetic stimulation should not alter firing rates, but only prevent the rhythmic firing of interneurons.

### Conclusions

After more than eight decades of research, gamma remains a mysterious and fascinating rhythm. We have extensively reviewed its mechanisms and putative functional role for perception, cognition, and behavior. We argue that progress towards understanding gamma’s function will accelerate, as we are starting to understand its true, transient nature and with the advent of powerful estimation tools that are able to correctly quantify its presence in neuronal signals. More recently, gamma entrainment using various stimulation techniques is finding its way as a therapeutic tool, with important applications in the treatment of AD. A picture starts to emerge where endogenous gamma oscillations, supported by the interneuronal machinery, may have an essential role in the maintenance of healthy circuits by means of fluctuating neurovascular coupling. We believe that the greatest discoveries regarding the multifaceted roles of gamma oscillations in brain function are about to be revealed in the near future, which may be closer than we think.

## Materials and methods

Data shown in Figures 1 and 3 were recorded using extracellular in vivo electrophysiology in mice and high-density EEG in humans.

### Experiments in mice with electrophysiology

Two types of experiments were conducted to test GENUS therapy, utilizing awake and anesthetized mice. All procedures complied with the guidelines of the European Communities Council Directive of 22/09/2010 (2010/63/UE) and were approved by local ethical committee (3/CE/02.11.2018) and the National Veterinary Authority (ANSVSA; 147/04.12.2018). Experiments were conducted on adult C57BL/6J mice housed in littermate groups of maximum three, in a controlled environment with temperature in the range of 21–23°C, 60% humidity, and under a light/dark cycle of 12/12 hr. Standard food and water were available ad libitum.

All surgical procedures were performed on anaesthetized animals using isoflurane (5% for induction, 2–2.5% for maintenance). Briefly, a circular craniotomy of 1 mm was performed to either implant electrodes or insert ASSY E-1 silicon (Cambridge NeuroTech) probes (for chronic and acute recordings, respectively). Electrophysiological data was recorded from the left visual cortex of the animal (0–0.5 mm anterior to lambda, 2–2.5 lateral to midline) at 32 kSamples /s during flicker stimulation. Flicker was delivered monocularly using a custom-made LED panel, for 6 s, @40 Hz, 50% duty cycle. Local field potentials were obtained by filtering the signal first with a low-pass filter @300 Hz, followed by a downsampling to 1 kHz, and high-pass filtering @0.1 Hz. Line noise and its harmonics were removed by notch filters @50 and @100 Hz.

### Experiments in humans using EEG

EEG data was obtained from a healthy human volunteer. All experimental protocols were approved by the Local Ethics Committee (approval 1/CE/08.01.2018) and data was collected in compliance with relevant legislation, that is Directive (EU) 2016/680 and Romanian Law 190/2018. Written consent was obtained prior to the experiment. EEG data was recorded @1024 samples/s using a high-density cap (128 electrodes – Biosemi ActiveTwo) from a healthy subject during a resting-state protocol with eyes closed. The data was subsequently band-pass filtered in a range of 0.1–200 Hz.
